# Evolutionary perspective of cancer: myth, metaphors, and reality

**DOI:** 10.1111/eva.12265

**Published:** 2015-05-27

**Authors:** Audrey Arnal, Beata Ujvari, Bernard Crespi, Robert A Gatenby, Tazzio Tissot, Marion Vittecoq, Paul W Ewald, Andreu Casali, Hugo Ducasse, Camille Jacqueline, Dorothée Missé, François Renaud, Benjamin Roche, Frédéric Thomas

**Affiliations:** 1 MIVEGEC, UMR IRD/CNRS/UM 5290Montpellier Cedex 5, France; 2 CREECMontpellier Cedex 5, France; 3 Centre for Integrative Ecology, School of Life and Environmental Sciences, Deakin UniversityWaurn Ponds, Vic., Australia; 4 Department of Biological Sciences, Simon Fraser UniversityBurnaby, BC, Canada; 5 Department of Radiology, H. Lee Moffitt Cancer Center & Research InstituteTampa, FL, USA; 6 Centre de Recherche de la Tour du Valat, ArlesFrance; 7 Department of Biology and the Program on Disease Evolution, University of LouisvilleLouisville, KY, USA; 8 Institute for Research in Biomedicine (IRB Barcelona)Barcelona, Spain; 9 International Center for Mathematical and Computational Modelling of Complex Systems (UMI IRD/UPMC UMMISCO)Bondy Cedex, France

**Keywords:** adaptationism, cancer, evolutionary processes

## Abstract

The evolutionary perspective of cancer (which origins and dynamics result from evolutionary processes) has gained significant international recognition over the past decade and generated a wave of enthusiasm among researchers. In this context, several authors proposed that insights into evolutionary and adaptation dynamics of cancers can be gained by studying the evolutionary strategies of organisms. Although this reasoning is fundamentally correct, in our opinion, it contains a potential risk of excessive adaptationism, potentially leading to the suggestion of complex adaptations that are unlikely to evolve among cancerous cells. For example, the ability of recognizing related conspecifics and adjusting accordingly behaviors as in certain free-living species appears unlikely in cancer. Indeed, despite their rapid evolutionary rate, malignant cells are under selective pressures for their altered lifestyle for only few decades. In addition, even though cancer cells can theoretically display highly sophisticated adaptive responses, it would be crucial to determine the frequency of their occurrence in patients with cancer, before therapeutic applications can be considered. Scientists who try to explain oncogenesis will need in the future to critically evaluate the metaphorical comparison of selective processes affecting cancerous cells with those affecting organisms. This approach seems essential for the applications of evolutionary biology to understand the origin of cancers, with prophylactic and therapeutic applications.

## Introduction

Following the pioneer works of Cairns (1975) and Nowell (1976), cancer is perceived as a phenomenon whose origins and dynamics result from evolutionary processes (Cairns 1975; Nowell 1976; Merlo et al. 2006; Greaves and Maley 2012; Thomas et al. 2013). Placing cancer in an evolutionary ecology landscape is not a semantic problem, but rather a necessity to understand the origins and progression of cancer with the ultimate aim of developing ways to control neoplastic progression and, most importantly, to prevent cancer and improve therapy when cancer occurs (Aktipis and Nesse 2013; Thomas et al. 2013). Because cancer cells abort their altruistic behavior in favor of a selfish life history strategy (that may eventually involve clonal cooperation), their evolution becomes, at least partially, governed by the same rules that apply to any autonomous entity prioritizing to maximize its own individual fitness. Several authors proposed that insights into evolutionary and adaptation dynamics of cancers can thus be gained by studying the evolutionary strategies of organisms (Deisboeck and Couzin 2009; Lambert et al. 2011; Ben-Jacob et al. 2012; Sprouffske et al. 2012; Korolev et al. 2014). Although this reasoning is fundamentally correct, in our opinion, it contains a potential risk of excessive adaptationism, potentially leading to the suggestion of complex adaptations that are unlikely to evolve among cancerous cells. Determining the limits of adaptation resulting from oncogenic selection (*sensu* Ewald and Swain Ewald 2013), and more generally the differences (as well as similarities) between cancer cell and organismal evolution, is fundamental to the applications of evolutionary biology to carcinogenesis and has direct implications for therapies designed to thwart cancer cell proliferation.

What are the primary differences between cancers and organisms, with regard to evolution and adaptation? First, cancer is an ancestral disease that probably developed almost immediately following the transition from unicellular to metazoan life, about one billion years ago (Domazet-Loso and Tautz 2010), but each cancer must ‘reinvent the wheel’ because their evolutionary products die within the host. Malignant cells are, at the best, under selective pressures for their altered lifestyle for only few decades, and a few dozen or hundreds of cell generations, and even less when the cancer itself reduces the lifespan of its host; for example, only 45 generations of replication are required, in principle, to go from a single cell to the 35–40 trillion cells in the human body. In this context, despite the rapid evolution of malignant cells, not all adaptive responses observed in (rapidly) reproducing unicellular organisms exposed to natural selection over tens of thousands to millions of years can be applied to cancer cells with short life histories. For instance, it has been argued that relatedness within tumors should influence cell decision to migrate (metastasize) and/or to locally cooperate (Deisboeck and Couzin 2009; Taylor et al. 2013). Such behavioral responses indeed exist in some animal species and microbes (West et al. 2006) to reduce competition and/or to promote the fitness of related individuals (Kawata 1987; Le Galliard et al. 2003; Moore et al. 2006). However, these life history strategies are the result of Darwinian evolution occurring over thousands and/or millions of years, not over mere decades. Unless ancestral heritable traits acquired prior to multicellularity are reactivated in cancerous cells, it is very unlikely that malignant cells would be able to display adaptive responses necessitating the ability of recognizing related conspecifics and adopting accordingly behaviors that depends on the kin context. Additional examples arise from the multistep process of metastasis. Studies have been arguing that the production and dissemination of metastatic cells should be counter selected at the initiation and early stages of tumors due to local resource availability (the selection should favor cells resistant to anoikis (programmed cell death) and contact inhibition, but with no migratory potential (Gatenby and Gillies 2008). At later stages when damage to the tumor accommodating organ significantly restricts resource availability, tumor cells with increased motility should have selective advantage (and higher fitness) despite the cost of most migrating cells dying without establishing a new colony (Merlo et al. 2006). However, recent studies challenge the traditional view of a late acquisition of metastatic potential and instead propose that tumor cells acquire the motile phenotype early in tumorigenesis (Eyles et al. 2010) as a result of selection favoring expansion of primary tumors. Pathologic cell mobility could indeed contribute significantly not only to metastasis but also to primary tumor growth (cancer self-seeding theory (Norton and Massagué 2006)), but the pathways to the self-seeding that the primary tumor will take depends on the cues and concomitant selective forces of tumor microenvironment. Welcoming nutrient rich or hostile-depleted primary tumor site will result in different outcomes (i) dislodging, then reattaching in/at the primary site, (ii) dislodging, circulation in blood stream then reattachment in/at the primary site, (iii) dislodging, circulation in blood stream then reattachment in a novel (metastatic) site (Norton and Massagué 2006).

Variability in temporal resource quality generating different selection pressures, and resulting in phenotypic divergence, including sympatric speciation, has been observed in numerous species (Ehlinger 1990; Spinks et al. 2000; Garant et al. 2005). However, in our mind, the relevance of these examples to cancer remains questionable because they involve adaptive context-dependent behaviors that result from long-term evolutionary processes, at least more than a few years or decades. The extent to which the context-dependent behaviors can be favored during oncogenic selection remains to be determined. Second, cancer cell evolution through oncogenic selection involves different mechanisms for the generation of genetic and phenotypic variability than organismal evolution: cancer cell lineages are asexual, they undergo very high rates of mutation often due to genomic instabilities, and the mutations and epimutations generated most commonly involve losses of gene functions and large alterations to gene dosages, rather than the small alterations to functions and dosages that appear to typify adaptive organismal evolution. Organismal adaptations often involve changes to cellular regulation that decrease cellular reproduction (e.g., through cell cycle arrest) and survival (e.g., through apoptosis) when such regulation increases the evolutionary fitness of the organism; selection during oncogenesis, however, typically favors abrogations of these regulatory adaptations (Ewald and Swain Ewald 2013). Selection during oncogenesis is therefore commonly destructive of organismal adaptations. Because destructive mutations are more common than mutations that generate or improve sophisticated biological mechanisms, mutation-driven evolution of cancer is feasible over short periods of time. The genomic variability of cancer cells, which may be due to mutagenic microenvironments favoring deficiencies in DNA repair (Breivik 2011), is among the causes of both malignancy itself and failures of chemotherapy due to evolved resistance (Merlo et al. 2006). Given that most mutations are deleterious to cell survival or replication, especially high variability must by first principles reduce the probability, precision, and sustainability of adaptive evolution in the context of complex fits to the environment. A possible example could be the neovascularization in cancer which involves the evolution of structures that are similar to blood vessels but less effective at transport because of aberrant angioarchitecture (Nagy et al. 2010). Larger mutations, as expected under genomic instabilities, are also expected to be less likely to generate adaptive change (Orr 2005). Asexual reproduction will also prevent or delay the coincidence of adaptive gene combinations within individual cells and thus should, in theory, hinder or delay the evolution of adaptations that rely on epistatic effects (De Visser and Elena 2007). Cancer cell populations surmount such population-genetic challenges, in part, through their very large population sizes, which greatly increase the scope for rare, favored alterations and reduce the efficacy of drift.

Contagious cancers [Tasmanian devil facial tumor disease (DFTD) and canine transmissible venereal tumor (CTVT) (Morgia et al. 2006; Murchison 2009)] present unique exceptions to the expectation, based on these considerations, of reduced adaptive evolution in cancer cells. In such cancers, fitness is not restricted by the death of the host, and the transmission of these clonal cancer cell lineages ensures their survival even after the carrying animal succumbs to the disease. CTVT and DFTD are the two oldest naturally occurring cancer cell lines, appearing approximately 11 000 and 20 years ago, respectively (Hawkins et al. 2006; Murchison et al. 2014). The evolutionary history of these cancers has allowed the development, pursuance, and implementation of highly elaborate adaptive strategies that maintain reproductive potential in the hostile micro- (stroma) and macro- (host's genotype) environment of their canine and devil hosts.

Finally, even though cancer cells can theoretically display highly sophisticated adaptive responses, it would be crucial to determine the frequency of their occurrence in patients with cancer, before therapeutic applications can be considered.

The extent to which selection akin to Darwinian evolution occurs in tumors is an old debate (Tarin et al. 2005; Scheel et al. 2007; Talmadge 2007); while it undoubtedly contributes to cancer development and progression (e.g., resistance to therapies), certain adaptive traits including the metastatic cascade could also be explained by phenotypic plasticity rather than selection (Scheel et al. 2007; Gatenby and Gillies 2008; Gatenby et al. 2011). Although the role of phenotypic plasticity in creating pathogenic cell motility is now a generally accepted and well-supported concept (Scheel et al. 2007; Gatenby and Gillies 2008; Gatenby et al. 2011), restrictively using Darwin's theories to explain metastasis would remain a misconception. Therefore, we think that it is important to add and emphasize the risk of excessive adaptationism applied to cancer evolution, and we hope to stimulate additional debate in this topic. Clearly, further work, especially theoretical models, based in population genetics, genomics, and the evolution of adaptation, is necessary to properly address this point.

The evolutionary perspective on cancer has gained significant international recognition over the past decade and, as all novel scientific paradigms, generated a wave of enthusiasm among researchers. The heightened interest is understandable as somatic cellular selection and evolution indeed offer an elegant adaptive explanation for carcinogenesis with its many manifestations (neoangiogenesis, evasion of the immune system, metastasis, and resistance to therapies). However, scientists who try to explain oncogenesis will need to critically evaluate the metaphorical comparison of selective processes affecting cancerous cells with those affecting organisms—both similarities and differences need to be carefully considered. This approach seems essential for the applications of evolutionary biology to understand the origin of cancers, to control neoplastic progression, and to improve therapies.

## Competing interests

The authors declare no conflict of interest.

## Authors' contributions

AA, BU, BC, RAG, MV, PE, AC, FR, BR, and FT were involved in the drafting of the article. All the co-authors revised the content of the manuscript and approved this version for publication.
